# Association of Pretreatment Serum Indirect Bilirubin Levels With Prognostic and Therapeutic Value in Patients With Newly Diagnosed Acute Myeloid Leukemia

**DOI:** 10.1002/cam4.70572

**Published:** 2025-01-27

**Authors:** Chunfang Kong, Linhui Hu, Ling Zhang, Hongbo Cheng, Qilin Lu, Anna Li, Bo Ke, Wenting Cui, Huixia Zhang, Mei Wu, Qingqing Zhu, Chenghao Jin, Li Yu

**Affiliations:** ^1^ Department of Hematology The Second Affiliated Hospital, Jiangxi Medical College, Nanchang University Nanchang Jiangxi China; ^2^ Department of Hematology Jiangxi Provincial People's Hospital, the First Affiliated Hospital of Nanchang Medical College Nanchang Jiangxi China; ^3^ Jiangxi Provincial Key Laboratory of Hematological Diseases Nanchang Jiangxi China; ^4^ National Clinical Research Center for Hematologic Diseases The First Affiliated Hospital of Soochow University Nanjing China; ^5^ Department of Hematology The First People's Hospital of Jiujiang City Jiujiang Jiangxi China

**Keywords:** acute myeloid leukemia, indirect bilirubin, prognosis

## Abstract

**Background:**

Bilirubin has anti‐inflammatory, antioxidant, and anti‐cancer properties, with an inverse relationship between its levels and cancer risk and prognosis. However, the prognostic value of serum bilirubin in acute myeloid leukemia (AML) remains uncertain.

**Methods:**

This retrospective study analyzed pretreatment serum total bilirubin (TBIL), direct bilirubin (DBIL), and indirect bilirubin (IBIL) in 284 AML patients and 316 healthy controls. The prognostic significance of serum bilirubin levels was determined using the Kaplan–Meier method and Cox proportional hazards model.

**Results:**

Pretreatment TBIL and IBIL levels were significantly lower in AML patients compared to controls. TBIL and IBIL levels were significantly higher in the CR/CRh/CRi group than in the non‐CR/CRh/CRi group and increased significantly after chemotherapy. Elevated pretreatment TBIL and IBIL were associated with longer overall survival (OS) (*p* < 0.05) and progression‐free survival (PFS) (*p* < 0.05). Pretreatment IBIL was an independent prognostic factor for OS (hazard ratio [HR], 0.47; 95% confidence interval [CI] 0.28–0.79; *p* < 0.05) and PFS (HR, 0.53; 95% CI 0.33–0.85; *p* < 0.05).

**Conclusion:**

Elevated pretreatment IBIL levels are correlated with improved OS and PFS, acting as an independent favorable prognostic indicator for AML.

## 1 Introduction

Acute myeloid leukemia (AML) is a highly heterogeneous hematological malignancy characterized by uncontrolled proliferation of myeloid stem cell precursors of the myeloid lineage [1]. Over the past few decades, advances in intensive chemotherapy, allogeneic hematopoietic stem cell transplantation, demethylating agents in combination with venetoclax, chemotherapy targeting AML driver gene mutations, and supportive therapy significantly improved the survival of AML patients. However, a considerable number of patients still face poor prognosis and short survival, particularly in elderly AML patients aged 65 years and older [2, 3]. At present, genetic abnormalities such as chromosomal aberrations and gene mutations are considered the most powerful prognostic information [4]. These prognostic markers are used to determine prognosis and guide treatment decisions. At present, AML stratification is primarily based on genetic profiling; however, beyond genetic stratification, there is still an urgent need to explore and evaluate new factors affecting the risk and prognosis of AML patients to improve their survival outcomes.

Bilirubin is the final product of heme catabolism, which is metabolized in the liver and conjugated to form water‐soluble direct bilirubin, which is then secreted into bile [5]. Unconjugated bilirubin, also known as IBIL, accounts for over 80% of total bilirubin. Although previously considered physiologically inert or even harmful, recent studies [6, 7, 8] have revealed that IBIL possesses significant anti‐inflammatory, antioxidant, and anti‐cancer effects. An inverse association between the level of IBIL and cancer risks and prognosis has been suggested in colorectal cancer [9], lung cancer [10], osteosarcoma [11], nasopharyngeal carcinoma [12, 13], and ovarian cancer [14]. Additionally, in vitro and in vivo studies [11, 13, 15, 16, 17, 18, 19] have confirmed that IBIL suppresses the growth of several cancer types through mechanisms such as inducing apoptosis, arresting cell cycle, inhibiting invasion and metastasis, exerting antioxidant and anti‐inflammatory functions, and modulating signaling pathways. Despite these findings, the relationship between IBIL levels and the survival of AML patients remains unexplored.

In this retrospective study, we aimed to investigate the impact of serum bilirubin levels at the time of AML diagnosis on patient prognosis and treatment response. Our objective was to determine whether these bilirubin fractions have a protective effect against AML and whether they could function as predictive biomarkers for AML patient survival and prognosis.

## 2 Methods

### 2.1 Patients and Methods

Between January 2012 and May 2023, 284 eligible patients diagnosed with AML at Jiangxi Provincial People's Hospital (The First Affiliated Hospital of Nanchang Medical College) were enrolled in this retrospective study as the AML group. In total, 316 healthy individuals matched in age and gender with the AML group were recruited from the physical examination department of the same hospital as the control group (Table S1). All data were obtained in accordance with the Declaration of Helsinki. This study was conducted with the approval of the Ethics Committee of Jiangxi Provincial People's Hospital (2024[66]).

The inclusion criteria for AML patients in this study were as follows: (1) cases defined as AML according to the World Health Organization (WHO) 2016 criteria [20]; (2) newly diagnosed AML without any prior anti‐cancer treatment. Patients were excluded from the clinical analysis if they had (1) acute promyelocytic leukemia; (2) severe cardiovascular, renal, hepatobiliary, or pancreatic diseases; or (3) elevated liver function test parameters (aspartate aminotransferase levels exceeding 40 IU/L, alanine aminotransferase levels exceeding 50 IU/L).

### 2.2 Data Collection and Follow‐Up

The clinical data of the subjects were obtained from electronic medical records. The collected information included age, gender, BMI (body mass index), bone marrow cytomorphology, bone marrow blasts, bone marrow erythrocytes, chromosome karyotype, immune typing, fusion genes, gene mutations, white blood cell (WBC) count, red blood cell (RBC) count, hemoglobin (HGB) level, platelet (PLT) count, prothrombin time (PT), activated partial thromboplastin time (APTT), fibrinogen (FIB‐C), total protein (TP), albumin (ALB), globulin (GLB), TBIL, DBIL, IBIL, serum creatinine (Scr), blood urea nitrogen (BUN), uric acid (UA), lactate dehydrogenase (LDH), triglycerides (TG), total cholesterol (TC), low‐density lipoprotein cholesterol (LDL‐C), and high‐density lipoprotein cholesterol (HDL‐C) at the time of AML diagnosis, along with the levels of TBIL, DBIL, and IBIL after the last chemotherapy or before recurrence.

Hematologic responses, including complete remission (CR), complete remission with partial hematological recovery (CRh), complete remission with incomplete hematologic recovery (CRi), overall survival (OS), and progression‐free survival (PFS), were mainly obtained through hospital records and/or telephone surveys. The evaluation endpoints were defined as follows: CR was defined as bone marrow blast cells < 5%, absence of circulating blasts and extramedullary diseases, absolute neutrophil count (ANC) ≥ 1.0 × 10^9^/L, and PLT ≥ 100 × 10^9^/L; CRh was defined as ANC ≥ 0.5 × 10^9^/L and PLT ≥ 50 × 10^9^/L, with all other CR criteria met; CRi was defined as meeting all CR criteria except residual neutropenia (ANC < 1.0 × 10^9^/L) or thrombocytopenia (PLT < 100 × 10^9^/L) [4]. OS was defined as the time interval from enrollment to death from any cause, while PFS was defined as the time from enrollment to disease progression or death from any cause. The last follow‐up was conducted in July 2024.

### 2.3 Statistical Analysis

Statistical analyses were performed using SPSS 23.0 software, GraphPad Prism version 7.0, and the R programming language (R Core Team, Vienna, Austria). Receiver operating characteristic (ROC) curves were used to determine the optimal cutoff values for TBIL, DBIL, and IBIL. Categorical variables were compared using the chi‐square test, while continuous variables were analyzed using the *t*‐test for normally distributed data or nonparametric tests for non‐normally distributed data. Kaplan–Meier (K–M) curves were used to estimate survival probabilities, and the log‐rank test was employed to compare survival differences between groups. The Cox proportional hazards model was used to identify independent risk factors through multivariate analyses. Nomograms and restricted cubic spline plots were generated using R. Statistical significance was defined as two‐tailed *p* values < 0.05.

## 3 Results

### 3.1 The Difference Between AML and Health Controls

**FIGURE 1:**
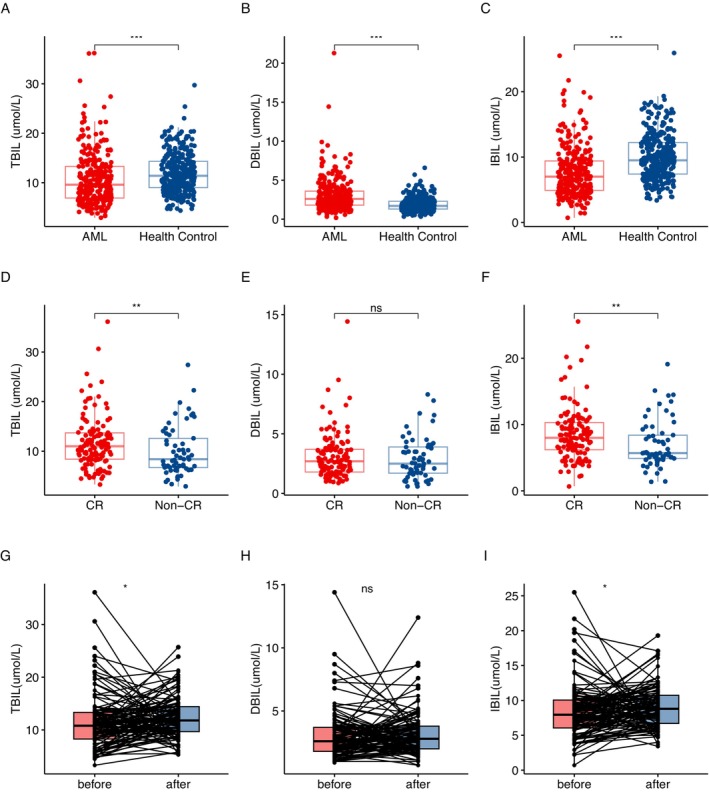
Comparison of bilirubin levels between AML patients and healthy controls, CR/CRh/CRi and non‐CR/CRh/CRi group, and comparison of bilirubin levels before and after treatment in CR/CRh/CRi group. (A) TBIL was significantly lower in AML patients than in healthy controls; (B) DBIL was significantly higher in AML patients than in healthy controls; (C) IBIL was significantly lower in AML patients than in healthy controls; (D) TBIL level was significantly higher in CR/CRh/CRi group than in non‐CR/CRh/CRi group; (E) DBIL level was not different between CR/CRh/CRi group and non‐CR/CRh/CRi group; (F) IBIL was significantly higher in CR/CRh/CRi group than in non‐CR/CRh/CRi group; (G) TBIL was significantly increased in the CR/CRh/CRi group after chemotherapy; (H) DBIL did not show a significant increase after chemotherapy in CR/CRh/CRi group; (I) IBIL was significantly increased in the CR/CRh/CRi group after chemotherapy.

Based on the inclusion and exclusion criteria, a total of 284 patients were recruited into the study. Among them, 154 (54%) were male, with a median age of 56 years (range: 13–89 years). The healthy control group consisted of 316 healthy individuals with matched age and sex characteristics to the AML cohort (Table S1). We compared the levels of bilirubin between participants in the AML group and those in the healthy control group. The serum TBIL levels in AML patients (Figure 1A *p* < 0.05), including IBIL (Figure 1C *p* < 0.05), were significantly lower than those in healthy individuals. In contrast, the DBIL levels were significantly higher in the AML group compared to the healthy control group (Figure 1B *p* < 0.05).

### 3.2 Relationships Between Bilirubin and Clinical Factors

The ROC curve analysis (Figure S1) determined optimal cutoff values for TBIL, DBIL, and IBIL at 8.55, 2.15, and 6.35 μmol/L, respectively. High TBIL, DBIL, and IBIL levels were observed in 59.2%, 64.8%, and 57.4% of patients. Significant differences were found between high and low TBIL groups in blasts (BM), erythrocytes (BM), and ALB levels (*p* < 0.05, Table 1). DBIL levels correlated with blasts (BM), erythrocytes (BM), APTT, TG, TC, and LDL‐C (*p* < 0.05, Table 1). IBIL levels were associated with erythrocytes (BM), RBC, HGB, PLT, and ALB (*p* < 0.05). Other clinical features showed no significant differences (*p* > 0.05, Table 1).

**TABLE 1:** The clinical characteristics of patients with AML based on bilirubin.

Variable	TBIL			DBIL			IBIL		
	Low	High	*p*	Low	High	*p*	Low	High	*p*
Total	116	168		100	184		121	163	
Age, y	55.0 (43.0–65.8)	57.0 (37.0–66.0)	0.874	54.0 (41.3–65.0)	57.0 (37.0–67.0)	0.666	57.0 (42.5–67.0)	56.0 (37.0–66.0)	0.435
Male	58 (50.0)	96 (57.1)	0.235	48 (48.0)	106 (57.6)	0.121	60 (49.6)	94 (57.7)	0.176
BMI, kg/m^2^	20.9 (19.4–22.8)	21.6 (19.6–23.9)	0.113	20.9 (19.4–23.1)	21.5 (19.5–23.8)	0.338	21.1 (19.3–23.3)	21.5 (19.6–23.8)	0.210
Blasts (BM), %	75.6 (53.5–87.4)	64.8 (46.1–82.4)	**0.018**	75.3 (54.7–87.4)	65.4 (46.6–83.1)	**0.028**	73.0 (52.4–85.4)	65.0 (46.8–83.5)	0.079
Erythrocytes (BM), %	1.8 (0.6–5.3)	5.8 (1.6–18.0)	**< 0.001**	2.5 (0.8–5.4)	5.0 (1.3–16.0)	**< 0.001**	1.8 (0.5–5.4)	5.9 (1.8–19.2)	**< 0.001**
WBC, ×10^9^/L	20.1 (4.7–49.5)	15.2 (3.5–51.2)	0.682	24.5 (4.9–56.4)	15.2 (3.6–47.0)	0.241	21.9 (4.7–49.4)	14.7 (3.5–52.5)	0.519
RBC, ×10^12^/L	2.11 (1.71–2.48)	2.22 (1.75–2.76)	0.100	2.15 ± 0.80	2.22 ± 0.78	0.447	2.07 ± 0.75	2.29 ± 0.81	**0.024**
HGB, g/L	68.0 (56.0–80.0)	72.0 (59.0–86.0)	0.112	69.5 ± 22.9	72.1 ± 20.2	0.310	67.6 ± 21.2	73.9 ± 20.9	**0.013**
PLT, ×10^9^/L	35.0 (15.3–72.5)	30.5 (17.0–52.0)	0.238	32.0 (15.0–66.3)	32.0 (17.3–56.3)	0.631	39.0 (16.5–78.0)	29.0 (17.0–49.0)	**0.034**
PT, s	13.1 (12.0–14.1)	13.2 (12.3–14.3)	0.303	13.2 (12.2–14.1)	13.2 (12.3–14.4)	0.545	13.1 (12.1–14.3)	13.2 (12.3–14.2)	0.900
APTT, s	29.9 (27.0–33.2)	29.5 (26.3–33.6)	0.509	28.0 (26.1–31.6)	30.2 (26.7–34.0)	**0.006**	30.5 (27.3–33.7)	28.7 (26.2–33.3)	0.069
FIB‐C, g/L	3.4 (2.5–4.3)	3.5 (2.7–4.5)	0.390	3.6 (2.9–4.4)	3.5 (2.6–4.5)	0.328	3.5 (2.5–4.3)	3.5 (2.7–4.5)	0.541
TP, g/L	63.9 (58.8–69.2)	64.1 (59.5–69.6)	0.480	64.7 (58.3–69.6)	63.9 (59.4–68.9)	0.782	63.8 (58.8–69.1)	64.1 (59.4–69.6)	0.401
ALB, g/L	34.4 ± 4.9	36.0 ± 4.9	**0.007**	35.1 ± 4.8	35.5 ± 5.1	0.563	34.3 ± 5.2	36.1 ± 4.7	**0.003**
GLB, g/L	28.1 (24.3–33.7)	27.5 (23.6–32.3)	0.213	28.9 (24.5–33.7)	27.5 (23.9–32.6)	0.223	27.8 (24.3–33.5)	27.5 (23.5–32.8)	0.272
Scr, μmol/L	68.5 (53.0–81.8)	72.0 (57.3–88.0)	0.086	68.0 (55.0–81.8)	71.5 (56.0–88.0)	0.232	70.0 (53.5–83.5)	71.0 (57.0–85.0)	0.354
BUN, mmol/L	5.0 (3.8–6.2)	4.8 (3.9–6.2)	0.667	4.8 (3.7–6.1)	4.9 (4.0–6.3)	0.294	4.9 (3.8–6.3)	4.8 (3.9–6.2)	0.770
UA, μmol/L	313.5 (231.8–389.8)	324.5 (254.3–425.8)	0.241	331.0 (249.3–405.8)	319.0 (238.3–415.3)	0.881	320.0 (224.0–394.0)	319.0 (256.0–415.0)	0.357
LDH, IU/L	403.0 (249.5–611.0)	412.0 (265.8–686.5)	0.396	380.5 (229.3–679.5)	417.0 (277.0–637.0)	0.388	407.0 (252.0–637.0)	408.5 (267.3–685.5)	0.498
TG, mmol/L	1.4 (1.0–1.7)	1.2 (1.0–1.7)	0.516	1.5 (1.1–1.9)	1.2 (1.0–1.7)	**0.034**	1.3 (1.0–1.6)	1.3 (1.0–1.8)	0.682
TC, mmol/L	3.0 (2.6–3.7)	3.0 (2.5–3.4)	0.499	3.3 (2.7–4.0)	2.9 (2.5–3.3)	**0.003**	2.8 (2.3–3.6)	3.1 (2.6–3.5)	0.186
HDL‐C, mmol/L	0.7 (0.5–0.8)	0.6 (0.5–0.8)	0.472	0.7 (0.6–0.9)	0.6 (0.5–0.8)	0.122	0.7 (0.5–0.7)	0.7 (0.5–0.8)	0.421
LDL‐C, mmol/L	1.7 (1.2–2.2)	1.6 (1.3–2.0)	0.436	1.8 (1.5–2.3)	1.5 (1.2–1.9)	**0.003**	1.6 (1.2–2.1)	1.6 (1.3–2.1)	0.518
ELN risk			0.190			0.071			0.249
Favorable	13 (20.9)	28 (32.6)		11 (17.7)	30 (34.9)		13 (20.6)	28 (32.9)	
Intermediate	12 (19.4)	19 (22.1)		15 (24.2)	16 (18.6)		14 (22.2)	17 (20.0)	
Adverse	37 (59.7)	39 (45.3)		36 (58.1)	40 (46.5)		36 (57.2)	40 (47.1)	

*Note:* Values were mean ± SD or median (IQR) for skewed variables, and numbers (proportions) for categorical variables. Bold values indicate that the clinical factors associated with high and low levels of TBIL, DBIL, and IBIL differ significantly from zero at the α = 0.05 level.

Abbreviations: ALB, albumin; AML, acute myeloid leukemia; APTT, activated partial thromboplastin time; BM, bone marrow; BMI, body mass index; BUN, blood urea nitrogen; DBIL, direct bilirubin; ELN, European leukemia Net; Fib‐C, fibrinogen; GLB, globulin; HDL‐C, high‐density lipoprotein cholesterol; HGB, hemoglobin; IBIL, indirect bilirubin; LDH, lactate dehydrogenase; LDL‐C, low‐density lipoprotein cholesterol; PLT, platelet; PT, prothrombin time; RBC, rhite blood cell; Scr, serum creatinine; TBIL, total bilirubin; TC, total cholesterol; TG, triglycerides; TP, total protein; UA, uric acid; WBC, white blood cell.

### 3.3 Relationship Between Serum Bilirubin Levels and Outcome

A total of 187 AML patients received at least one cycle of induction chemotherapy, and the efficacy was evaluated after one cycle of treatment. The relationship between the bilirubin levels at diagnosis and the curative effect in these patients was further analyzed. Patients who achieved CR/CRh/CRi exhibited significantly higher TBIL (Figure 1D *p* < 0.05) and IBIL (Figure 1F *p* < 0.05) levels at diagnosis compared to the non‐CR/CRh/CRi group (Table 2). In total, 143 patients who received two cycles of induction chemotherapy. Higher levels of TBIL (*p* < 0.05) and especially IBIL (*p* < 0.05) in AML patients were associated with achieving CR/CRh/CRi (Table 2). However, there was no significant difference in DBIL levels between the two groups (Table 2 and Figure 1E).

**TABLE 2:** The changes of serum bilirubin levels at diagnosis of AML in different therapeutic groups.

Variable	One cycle of chemotherapy (*N* = 187)			Two cycles of chemotherapy (*N* = 143)		
	CR/CRh/CRi (*N* = 126)	Non‐CR/CRh/CRi (*N* = 61)	*p*	CR/CRh/CRi (*N* = 125)	Non‐CR/CRh/CRi (*N* = 18)	*p*
TBIL, μmol/L	11.0 (8.4–13.7)	8.4 (6.6–13.0)	**0.008**	11.0 (8.1–13.8)	7.1 (6.3–12.4)	**0.050**
DBIIL, μmol/L	2.7 (1.8–3.8)	2.5 (1.7–3.9)	0.401	2.7 (1.8–3.8)	2.2 (1.6–4.2)	0.456
IBIL, μmol/L	8.2 (6.2–10.4)	5.7 (4.8–8.6)	**0.003**	8.0 (5.7–10.5)	5.1 (4.7–7.9)	**0.029**

*Note:* Values are median (IQR) for skewed variables. Bold values indicate that the levels of TBIL, DBIL, and IBIL between the CR/CRh/CRi group and the non‐CR/CRh/CRi group differ significantly from zero at the α = 0.05 level.

Abbreviations: AML, acute myeloid leukemia; CR, complete remission; CRh, complete remission with partial hematological recovery; CRi, complete remission with incomplete hematologic recovery; DBIL, direct bilirubin; IBIL, indirect bilirubin; TBIL, total bilirubin.

We conducted a paired comparison of bilirubin levels before and after treatment in CR/CRh/CRi group. We found a significant increase in TBIL (Figure 1G *p* < 0.05) and IBIL (Figure 1I *p* < 0.05) levels after chemotherapy. However, DBIL levels did not show a significant increase (Figure 1H *p* > 0.05).

### 3.4 Association Between Serum Bilirubin Levels and Survival in AML Cohort

**FIGURE 2:**
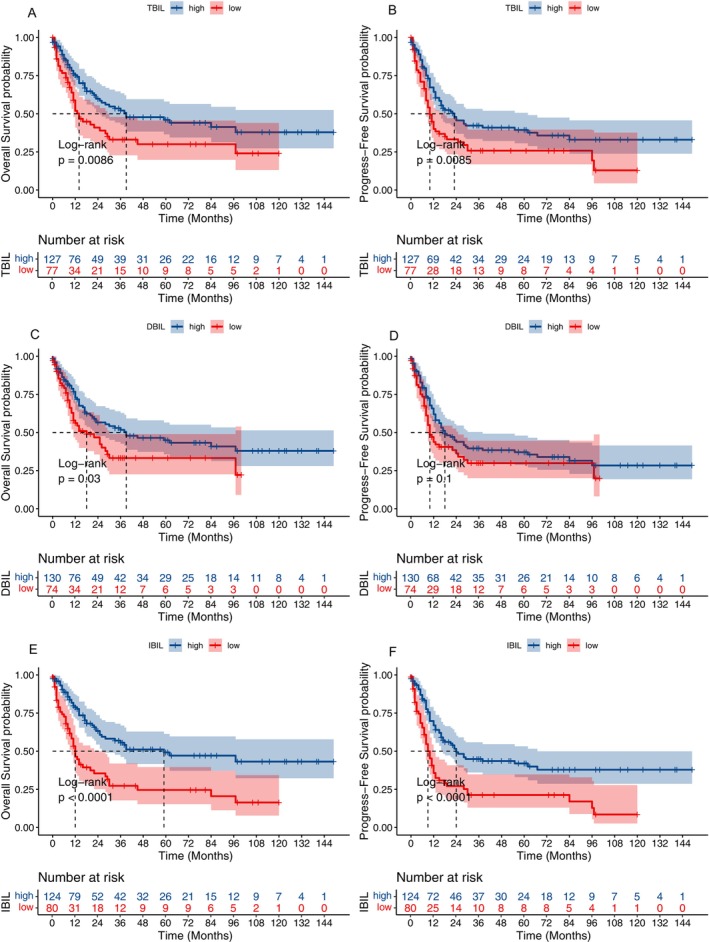
Kaplan–Meier curves for OS and PFS by bilirubin levels in patients with AML. (A) OS by TBIL levels; (B) PFS by TBIL levels; (C) OS by DBIL levels; (D) PFS by DBIL levels; (E) OS by IBIL levels; (F) PFS by IBIL levels.

The association between survival outcomes and serum bilirubin levels was examined in 204 AML patients who received at least one induction chemotherapy regimen. Kaplan–Meier curves revealed that AML patients with elevated levels of TBIL had better OS (*p* < 0.05, Figure 2A) and PFS (*p* < 0.05, Figure 2B). Moreover, AML patients with high levels of IBIL had markedly increased survival, both in terms of OS (*p* < 0.05; Figure 2E) and PFS (*p* < 0.05; Figure 2F). AML patients with high levels of DBIL had better OS (*p* < 0.05, Figure 2C) but not PFS (*p* > 0.05, Figure 2D).

### 3.5 Univariate and Multivariate Cox Regression Analyses of Prognostic Factors

To identify potential prognostic factors, univariate and multivariate Cox regression analyses were conducted on the OS and PFS of AML patients (Table 3). In the univariate analysis, age, blasts (BM), erythrocytes (BM), WBC count, TBIL, DBIL, IBIL, and ELN risk were significantly associated with OS (*p* < 0.05). In addition, the multivariate analysis showed that age (HR, 1.87; 95% CI 1.09–3.22; *p* < 0.05), IBIL (HR, 0.47; 95% CI 0.28–0.79; *p* < 0.05), and ELN risk (adverse vs. favorable, HR, 2.11; 95% CI 1.16–3.84; *p* < 0.05) were independent prognostic factors for patients with AML.

**TABLE 3:** Univariate and multivariate Cox hazard analysis for overall survival and progression‐free survival in AML.

Parameters	OS						PFS					
	Univariate analysis			Multivariate analysis			Univariate analysis			Multivariate analysis		
	HR	(95% CI)	*p*	HR	(95% CI)	*p*	HR	(95% CI)	*p*	HR	(95% CI)	*p*
Age, y			**0.001**			**0.024**			**0.001**			**0.031**
< 60	1	Reference		1	Reference		1	Reference		1	Reference	
≥ 60	2.02	(1.33, 3.08)		1.87	(1.09, 3.22)		1.99	(1.35, 2.93)		1.73	(1.05, 2.84)	
Sex			0.186						0.258			
Male	1	Reference					1	Reference				
Female	1.31	(0.88, 1.95)					1.24	(0.86, 1.78)				
Blast (BM), %			**0.018**			0.050			**0.117**			
< 88.13	1	Reference		1	Reference		1	Reference				
≥ 88.13	1.73	(1.10, 2.74)		1.75	(1.00, 3.08)		1.42	(0.92, 2.20)				
Erythrocyte (BM), %			**0.043**						**0.238**			
< 9.25	1	Reference					1	Reference				
≥ 9.25	0.61	(0.37, 0.99)					0.78	(0.51, 1.18)				
WBC count, ×10^9^/L			**0.022**						0.135			
< 100	1	Reference					1	Reference				
≥ 100	1.89	(1.10, 3.25)					1.49	(0.88, 2.50)				
HGB, g/L			0.280						0.674			
< 60	1	Reference					1	Reference				
≥ 60	0.79	(0.51, 1.21)					0.92	(0.61, 1.38)				
PLT count, ×10^9^/L			0.633						0.638			
< 30	1	Reference					1	Reference				
≥ 30	1.10	(0.74, 1.65)					1.09	(0.76, 1.58)				
ALB, g/L			0.788						0.829			
< 30	1	Reference					1	Reference				
≥ 30	0.92	(0.49, 1.72)					0.94	(0.53, 1.67)				
TBIL, μmol/L			**0.010**						**0.011**			
< 8.55	1	Reference					1	Reference				
≥ 8.55	0.59	(0.40, 0.88)					0.62	(0.43, 0.89)				
DBIL, μmol/L			**0.034**						0.108			
< 2.15	1	Reference					1	Reference				
≥ 2.15	0.64	(0.43, 0.97)					0.74	(0.50, 1.07)				
IBIL, μmol/L			**< 0.001**			**0.004**			**< 0.001**			**0.008**
< 6.35	1	Reference		1	Reference		1	Reference		1	Reference	
≥ 6.35	0.44	(0.30, 0.65)		0.47	(0.28, 0.79)		0.48	(0.33, 0.69)		0.53	(0.33, 0.85)	
ELN risk			**0.011**			**0.019**			**0.034**			**0.060**
Favorable	1	Reference		1	Reference		1	Reference		1	Reference	
Intermediate	1.10	(0.51, 2.36)	0.822	1.02	(0.46, 2.24)	0.965	1.57	(0.81, 3.06)	0.185	1.48	(0.75, 2.92)	0.253
Adverse	2.24	(1.24, 4.05)	**0.008**	2.11	(1.16, 3.84)	**0.015**	2.10	(1.20, 3.67)	**0.010**	1.97	(1.12, 3.46)	**0.018**

Abbreviations: ALB, albumin; AML, acute myeloid leukemia; BM, bone marrow; DBIL, direct bilirubin; ELN, European leukemia Net; HGB, Hemoglobin; IBIL, indirect bilirubin; OS, overall survival; PFS, progression‐free survival; PLT, platelet; TBIL, total bilirubin; WBC, white blood cell. Bold values indicate that the prognostic factors for OS or PFS in both univariate and multivariate COX regression analyses differ significantly from zero at the α = 0.05 level.

For PFS, age (*p* < 0.05), TBIL (*p* < 0.05), IBIL (*p* < 0.05), and ELN risk (*p* < 0.05) were statistically significant in the univariate Cox regression analysis. Multivariate analyses revealed that age (HR, 1.73; 95% CI 1.05–2.84; *p* < 0.05), IBIL (HR, 0.53; 95% CI 0.33–0.85; *p* < 0.05), and ELN risk (adverse vs. favorable, HR, 1.97; 95% CI 1.12–3.46; *p* < 0.05) were independent predictors of PFS (Table 3). These data suggest that serum IBIL levels serve as an independent prognostic factor for both OS and PFS in AML patients.

**FIGURE 3:**
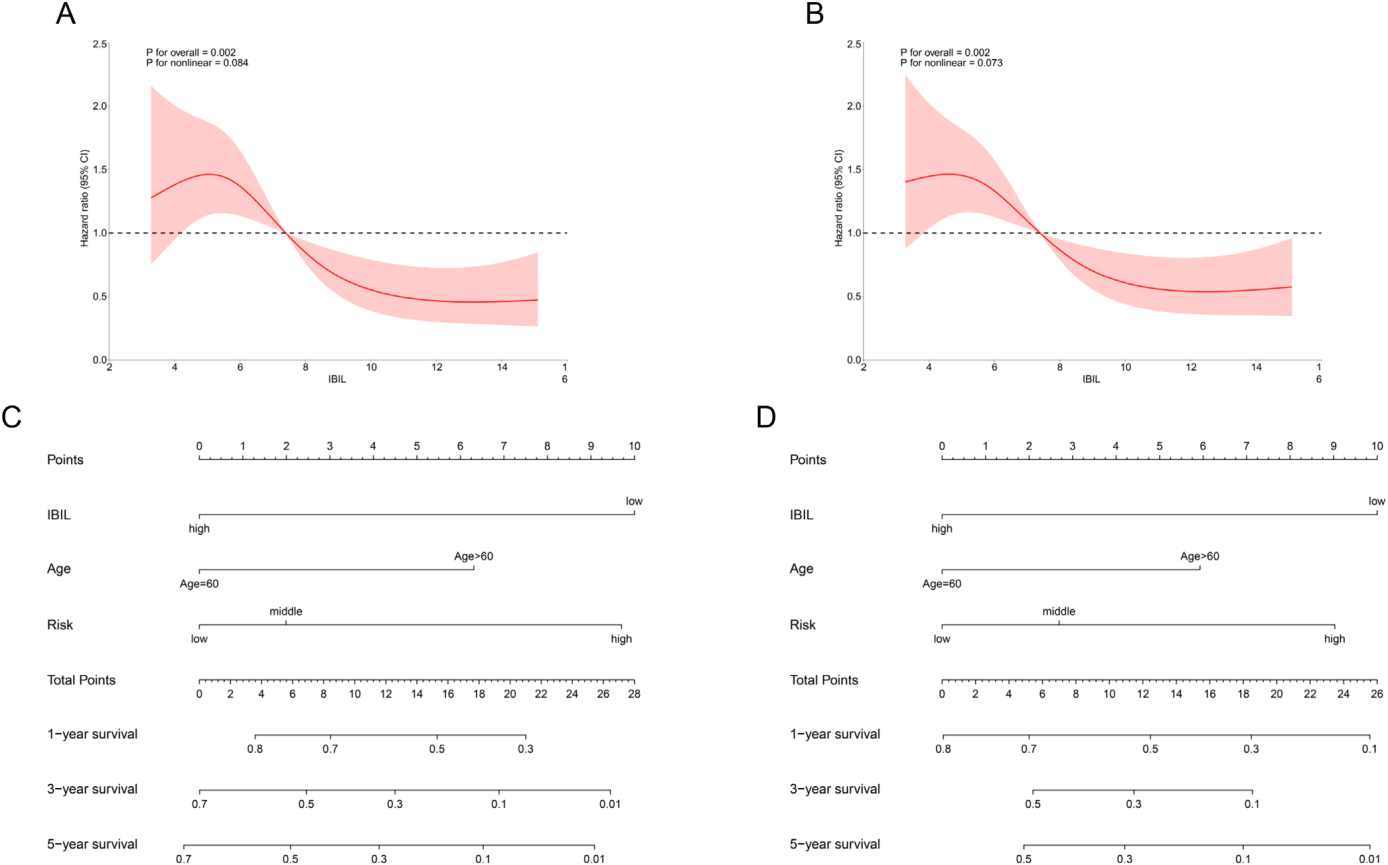
Restricted cubic splines and construction of the nomogram model. (A) Restricted cubic splines for the associations of IBIL with OS; (B) Restricted cubic splines for the associations of IBIL with PFS; (C) Nomogram predicting 1‐, 3‐ and 5‐year OS for patients with IBIL; (D) Nomogram predicting 1‐, 3‐ and 5‐year PFS for patients with IBIL.

We employed restricted cubic splines (RCS) to assess the potential nonlinear relationships between IBIL levels and both OS and PFS. The analysis revealed linear relationships in both the OS model (*p* for nonlinearity > 0.05, Figure 3A) and the PFS model (*p* for nonlinearity > 0.05, Figure 3B). Additionally, higher IBIL levels were significantly associated with a reduced risk of shorter OS (*p* < 0.05, Figure 3A) and PFS (*p* < 0.05, Figure 3B) in AML patients.

### 3.6 Construction of Prognostic Nomogram Based on IBIL

Based on the results of univariate and multivariate Cox regression analyses (Table 3), age, IBIL levels, and European LeukemiaNet (ELN) risk classification were incorporated into the prognostic nomograms for OS (Figure 3C) and PFS (Figure 3D). Age, IBIL levels, and ELN risk stratification are treated as independent variables, each quantified on a point scale. The total score is calculated by summing the points from all variables. To use the nomogram, the total number of points is plotted on the “total score” axis, and the predicted 1‐, 3‐, or 5‐year survival probability can be determined using the bottom scale.

## 4 Discussion

To the best of our knowledge, this study provides the first clinical evidence that patients with AML have significantly lower levels of TBIL and IBIL compared to healthy controls at initial diagnosis, while DBIL levels are significantly elevated. Moreover, AML patients with higher levels of TBIL and IBIL are more likely to achieve CR/CRh/CRi. Serum bilirubin levels significantly increase after chemotherapy in the CR/CRh/CRi group. Notably, elevated pretreatment TBIL and IBIL were associated with longer OS and PFS. Pretreatment IBIL was an independent prognostic factor for OS and PFS. Our findings suggest that IBIL serves as an independent favorable prognostic indicator for AML.

It is well known that AML is the most common type of leukemia in adults, the main clinical manifestations of AML include anemia, bleeding, and infection, making it a leading cause of hospitalization and death [21]. AML cells can remodel the bone marrow microenvironment to promote leukemia progression and inhibit normal hematopoiesis [22]. 80% of total bilirubin is derived from the breakdown of aging red blood cells, forming hemoglobin, which is catalyzed by heme oxygenase and biliverdin reductase to produce IBIL, and then transported to the liver by albumin and conjugated with glucuronic acid through UDP glucuronosyltransferase (UGT1A1) to form water‐soluble conjugated bilirubin, which is excreted into the bile [23, 24, 25]. Weisiger et al. [26] proposed that the affinity between human serum albumin and bilirubin is related to albumin concentration and buffer solution. It is well known that bilirubin levels are closely associated with liver function, erythrocyte catabolism, and ineffective erythropoiesis resulting from intramedullary erythroblast death [5]. In this study, all patients had normal liver function. However, AML patients may exhibit lower indirect bilirubin levels due to reduced erythrocyte mass in the peripheral blood and decreased bone marrow erythropoiesis. Lower bilirubin levels could indicate more extensive bone marrow infiltration by leukemic cells, leading to further suppression of erythropoiesis, which may reflect a more aggressive disease phenotype. Our study explored the association between bilirubin levels and clinical features, such as bone marrow blasts, bone marrow erythrocytes, peripheral RBC count, hemoglobin, and albumin. However, the absence of reticulocyte count data limited a more comprehensive analysis. The findings revealed that elevated TBIL, DBIL, and IBIL levels were linked to a decrease in bone marrow blasts and an increase in bone marrow erythrocytes. Moreover, higher IBIL levels were associated with increased albumin, RBC counts, and hemoglobin, potentially explaining the variations in bilirubin levels among AML patients.

In the past decades, multiple studies have demonstrated that bilirubin has previously unknown functions. It has now been established that bilirubin acts as a hormone, and moderately elevated levels are protective against metabolic and cardiovascular diseases, as well as tumors [6, 25, 27]. Many of the protective effects of bilirubin are attributed to its antioxidant [28, 29], anti‐inflammatory [30], and immunomodulatory properties [31]. Li et al. [10] showed that high levels of TBIL, DBIL, and IBIL were associated with longer OS, disease‐free survival, and distant metastasis‐free survival in patients with non‐small cell lung cancer who underwent curative resection. Xi et al. [14] have demonstrated that ovarian cancer patients with high preoperative TBIL (≥ 9.65 μmol/L) and IBIL (≥ 6.75 μmol/L) have longer OS and PFS. Multivariate analysis also showed that patients with high IBIL had significantly longer OS and PFS than those with low IBIL. In overall and gender‐stratified osteosarcoma patients, IBIL also functioned as an independent protective factor for both OS and PFS [11]. Currently, there is no research on the effect of bilirubin on AML. In our study, we found that IBIL is an independent prognostic factor for OS and PFS in AML patients. Additionally, patients with high levels of TBIL and IBIL are more likely to achieve CR/CRh/CRi. Moreover, serum TBIL and IBIL levels significantly increase after chemotherapy in the CR/CRh/CRi group. Our research indicates that IBIL plays a crucial role in AML.

In addition, the risk stratification of AML has undergone continuous reform and improvement. Initially, factors such as white blood cell count and age were the main considerations. In recent years, with the deepening understanding of the occurrence and development of AML, it has been found that the prognosis of AML patients is significantly correlated with chromosomal and genetic abnormalities [4]. However, patients with the same ELN risk may have different treatment responses and survival outcomes. The definition of risk stratification is limited by the times, and with the exploration of research, new prognostic indicators may be discovered, thus risk stratification has been continuously improving. Many nomograms have been developed for predicting survival, and factors such as age, ELN risk, and IBIL have been incorporated into our nomograms. Our results showed that these nomograms had a greater clinical application potential for predicting 1‐, 3‐, and 5‐year OS and PFS. Moreover, we also established restricted cubic splines and found a linear relationship between IBIL and AML survival prognosis risk. With the increase of bilirubin concentration, the risk of OS and PFS in AML patients is significantly reduced.

Although these results indicate that IBIL is an independent predictor of survival in AML patients and may be used as a useful tool for treatment planning decisions and even predicting the prognosis of AML, our study has several limitations. To begin with, selection bias cannot be ruled out in the retrospective data collection process. Furthermore, the inclusion period of patients is relatively long (from 2012 to 2023), and in the early stages, due to medical conditions, many patients had incomplete ELN prognostic staging. Lastly, this study consists of patient data from a single cancer center, and it is necessary to conduct large‐scale, multicenter research to validate our results.

## 5 Conclusion

In summary, this study confirmed that IBIL is an independent prognostic factor for AML patients. The report suggests that IBIL is a low‐cost and reliable tool for predicting the survival rate of AML patients, as well as a favorable drug target for improving AML treatment.

## Author Contributions

**Chunfang Kong:** conceptualization, methodology, project administration, validation, and writing the original draft. **Linhui Hu:** statistical analysis and validation. **Ling Zhang:** supervision and data curation. **Hongbo Cheng:** methodology and conceptualization. **Qilin Lu:** data curation. **Anna Li:** visualization and software. **Bo Ke:** visualization and investigation. **Wenting Cui:** supervision and data curation. **Huixia Zhang:** supervision and data curation. **Mei Wu:** validation and investigation. **Qingqing Zhu:** data curation and investigation. **Chenghao Jin:** review, editing, supervision, and funding acquisition. **Li Yu:** formal analysis, resources, supervision, review, editing, and funding acquisition.

## Ethics Statement

All the participants provided written informed consent. All procedures performed in studies were in accordance with the Declaration of Helsinki. This study was approved Medical Ethics Committee of Jiangxi Provincial People's Hospital, The First Affiliated Hospital of Nanchang Medical College, Jiangxi, China.

## Conflicts of Interest

The authors declare no conflicts of interest.

## Supporting information


**Figure S1.** Receiver operating characteristic (ROC) curves and area under curve (AUC) values. (A) ROC and AUC of the TBIL; (B) ROC and AUC of the DBIL; (C) ROC and AUC of the IBIL.

**Table S1.** The baseline characteristics of the study participants.

## Data Availability

The datasets used and/or analyzed during the current study are available from the corresponding author upon reasonable request.
