# Supplementary figures and images for: Association of Pretreatment Serum Indirect Bilirubin Levels With Prognostic and Therapeutic Value in Patients With Newly Diagnosed Acute Myeloid Leukemia
Source: Cancer Med. 2025 Jan 27;14(3):e70572. doi: 10.1002/cam4.70572 (PMC11770887; doi:10.1002/cam4.70572)

A

TBIL

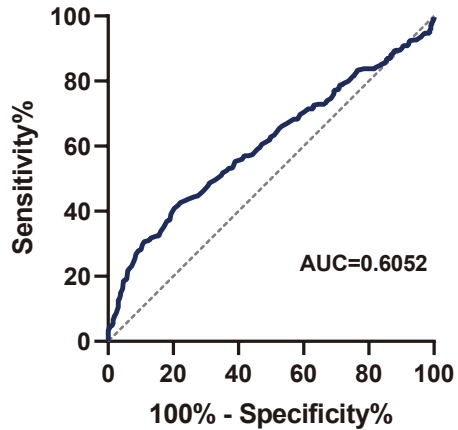

B

DBIL

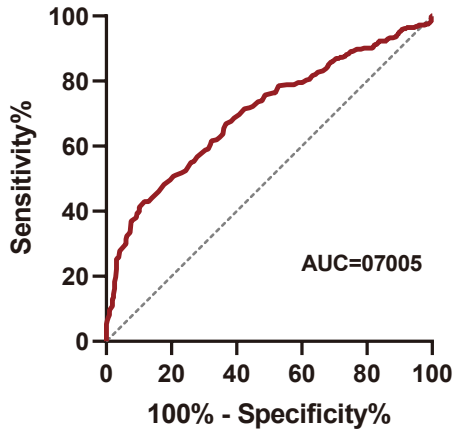

C

IBIL

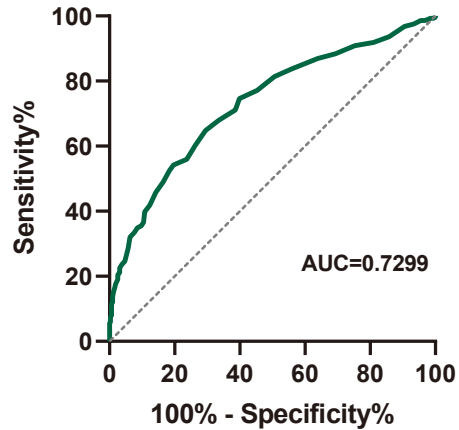

Supplement: Supplementary file 1 — Figure S1. Receiver operating characteristic (ROC) curves and area under curve (AUC) values. (A) ROC and AUC of the TBIL; (B) ROC and AUC of the DBIL; (C) ROC and AUC of the IBIL. [file CAM4-14-e70572-s002.pdf]
