# Supplementary material for: Association of Pretreatment Serum Indirect Bilirubin Levels With Prognostic and Therapeutic Value in Patients With Newly Diagnosed Acute Myeloid Leukemia
Source: Cancer Med. 2025 Jan 27;14(3):e70572. doi: 10.1002/cam4.70572 (PMC11770887; doi:10.1002/cam4.70572)
Supplement: Supplementary file 2 — Table S1. The baseline characteristics of the study participants. [file CAM4-14-e70572-s001.docx]

**Supplementary table 1. The baseline characteristics of the study participants**

| **Characteristics** | **AML**  **(N = 284)** | **Healthy controls**  **(N = 316)** | **P-value** |
| --- | --- | --- | --- |
| Age in years | 56.0 (40.0-66.0) | 50.0 (42.0-60.0) | 0.055 |
| Sex  Male, N (%) | 154 (54.2) | 162 (51.3) | 0.468 |
| Female, N (%) | 130 (45.8) | 154 (48.7) |  |
| TBIL, μmol/L | 9.6 (6.9-13.3) | 11.4 (90-14.4) | **< 0.001** |
| DBIL, μmol/L | 2.6 (1.8-3.6) | 1.7 (1.3-2.3) | **< 0.001** |
| IBIL, μmol/L | 7.0 (4.9-9.4) | 9.5 (7.4-12.3) | **< 0.001** |

Values were mean ± SD or median [IQR] for skewed variables, and numbers (proportions) for categorical variables.

Abbreviations: AML, acute myeloid leukemia; TBIL, total bilirubin; DBIL, direct bilirubin; IBIL, indirect bilirubin.
